# Two novel human NUMB isoforms provide a potential link between development and cancer

**DOI:** 10.1186/1749-8104-5-31

**Published:** 2010-12-01

**Authors:** Aldona Karaczyn, Mahmud Bani-Yaghoub, Roger Tremblay, Chris Kubu, Rebecca Cowling, Tamara L Adams, Igor Prudovsky, Douglas Spicer, Robert Friesel, Calvin Vary, Joseph M Verdi

**Affiliations:** 1 Center for Molecular Medicine, Maine Medical Center Research Institute, 81 Research Dr. Scarborough, ME 04074, USA; 2 Neurogenesis and Brain Repair, Institute for Biological Sciences, National Research Council of Canada, Bldg M-54, 1500 Montreal Road, Ottawa, ON, K1A 0R6, Canada; 3 Department of Cellular and Molecular Medicine, University of Ottawa, Ottawa, ON, K1H 8L6, Canada; 4 USB Pharmaceuticals, Cleveland, OH 44128, USA; 5 Samuel Lunenfeld Research Institute, Mount Sinai Hospital, Toronto, ON, M5G 1X5, Canada

## Abstract

We previously identified four functionally distinct human NUMB isoforms. Here, we report the identification of two additional isoforms and propose a link between the expression of these isoforms and cancer. These novel isoforms, NUMB5 and NUMB6, lack exon 10 and are expressed in cells known for polarity and migratory behavior, such as human amniotic fluid cells, glioblastoma and metastatic tumor cells. RT-PCR and luciferase assays demonstrate that NUMB5 and NUMB6 are less antagonistic to NOTCH signaling than other NUMB isoforms. Immunocytochemistry analyses show that NUMB5 and NUMB6 interact and complex with CDC42, vimentin and the CDC42 regulator IQGAP1 (IQ (motif) GTPase activating protein 1). Furthermore, the ectopic expression of NUMB5 and NUMB6 induces the formation of lamellipodia (NUMB5) and filopodia (NUMB6) in a CDC42- and RAC1-dependent manner. These results are complemented by *in vitro *and *in vivo *studies, demonstrating that NUMB5 and NUMB6 alter the migratory behavior of cells. Together, these novel isoforms may play a role in further understanding the NUMB function in development and cancer.

## Introduction

*Numb *was originally described as a mutation affecting the binary division of the sensory organ progenitor cells and a key player in neural cell fate decisions in *Drosophila *[1-4]. Loss of *Numb *function results in cells adopting non-neuronal fates, while ectopic Numb expression leads to additional neurons in *Drosophila *at the expense of other differentiated cell types. The cloning of mammalian *Numb *homologues [5-7] and the subsequent demonstration that these homologues affect mammalian differentiation outcomes [5-9] suggest an evolutionarily conserved function for Numb. These data are further supported by *Numb *knock-out phenotypes [10,11]. Although cell polarity is tightly regulated in mammalian development [12] and cancer [13], *Numb *mutations can lead to loss of cell polarity and alteration of cell fate in *Drosophila *[14]. However, unlike the invertebrate *numb *gene, the mammalian *Numb *gene is alternatively spliced, producing four functionally distinct protein isoforms, Numb1-4 [5,6,9,12,15]. In addition to its role in neurogenesis, *Numb *has also been considered a tumor suppressor gene by reciprocally regulating the function of Notch in carcinogenesis [13,16,17]. This notion originated from evidence that breast tumors and gliomas demonstrate altered levels of *Numb *and *Notch *mRNA [18-20]. However, the mechanisms by which Numb influences tumorigenicity and cancer remain to be determined and the exact mechanism by which Numb antagonizes Notch are still elusive. Recently, in an elegant series of experiments, McGill and McGlade [21] demonstrated that numb interactions with Notch result in an increase in Notch ubiquitination, resulting in lower levels of Notch protein and consequently attenuated downstream effects.

Here, we present evidence for the existence of at least two and potentially four novel human NUMB isoforms (NUMB5 and NUMB6 (NUMB5/6)) that arise from the alternative splicing of exon 10 of the mammalian *Numb *gene. These isoforms are transient and rare in normal tissues but are abundantly expressed in transformed and cancerous cells. NUMB5/6 have less antagonizing effects on Notch signaling than other NUMB proteins, as shown by RT-PCR and Notch transactivation studies. The over-expression of NUMB5/6 phenocopies the effects of Notch activation, leading to rapid cellular formations in cortical progenitors, N2a cells and HEK293 cells. Moreover, in opposition to the actions of previously described NUMB isoforms, and similar to the actions of activated Notch, over-expression of NUMB5/6 in differentiated cortical neurons restricts process length and increases process branching. Furthermore, NUMB5 and NUMB6 are co-localized with and regulated by CDC42 such that inhibiting CDC42 activity abrogates the formation of cell protrusions and migration. Finally, because of these interactions, NUMB5/6 have the capability to regulate cytoskeleton assembly and cell migration similarly to Notch, suggesting these novel isoforms are dominant negative versions of the NUMB1-4 isoforms.

## Results

### The cloning of NUMB5 and NUMB6

**Figure 1:**
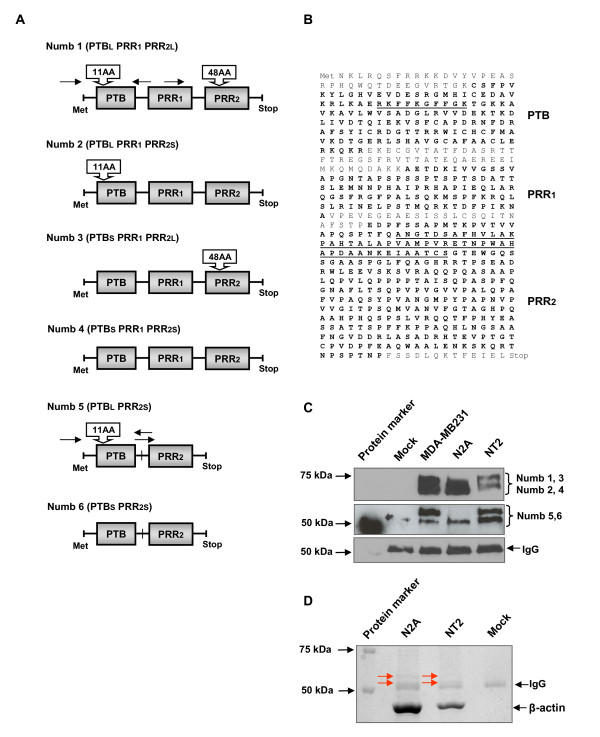
**Cloning of the novel human NUMB isoforms**. **(A**) The nomenclature and structure of novel (NUMB5 and NUMB6) and previously reported NUMB isoforms. NUMB5 lacks the proline rich region PRR1 and NUMB6 lacks both the phosphotyrosine binding domain and PRR2 inserts and PRR1. **(B) **The amino acid sequence of NUMB isoforms. The PTB domain, PRR1, and PRR2 are shown in bold and the PTB domain (11 amino acids long) and PRR (48 amino acids long) inserts are underlined. **(C) **Western blot detection of novel NUMB isoforms. The two fractions of novel NUMB proteins differed from generic NUMB isoforms by approximately 10 kDa, most likely representing two novel isoforms of NUMB, NUMB5 and NUMB6. **(D) **Coomassie blue stained protein bands used for mass spectrometry protein sequencing. Protein bands indicated by red arrows correspond to NUMB5 and NUMB6, respectively. β-actin fraction served as an internal control in mass spectral sequence analysis.

Our cloning of rat [6] and human [5] Numb homologues and the subsequent demonstration that these homologues direct mammalian neural stem cells toward a neuronal phenotype [5,6,22] suggest an evolutionarily conserved function for Numb and is consistent with recently published observations from Numb knock-out/ablation [10,11,23,24]. Unlike the invertebrate *numb *gene, the mammalian *Numb *gene was shown to be alternatively spliced, producing four functionally distinct Numb protein isoforms (Figure 1A). The well-established human teratocarcinoma NT2/D1 cells were utilized [25] to ascertain the expression pattern of human *NUMB *transcripts *in vitro *during neuronal differentiation. Similar to human embryonic stem and neural stem cells, NT2/D1 cells have the capacity to differentiate into neurons and astrocytes when cultured in the presence of retinoic acid [25-27]. To detect transient *NUMB *expression, cells were examined in the undifferentiated state and at time points from 4 days to 5 weeks of retinoic acid treatment using human *NUMB*-specific primers that annealed just 5' to the splice site contained within the phosphotyrosine binding (PTB) domain insertion site and 3' to the splice site within the proline rich region (PRR) [6] (Additional file 1). Surprisingly, in addition to the expected *NUMB *amplicons, smaller unexpected amplicons were observed during the differentiation process 4 days after retinoic acid treatment. The cloning and sequencing of these products revealed an in-frame *NUMB *cDNA that had a 294-nucleotide deletion located between the PTB domain and PRR splice sites (Figure 1B). It was determined that the deleted region corresponded to exon 10 of the published genomic *NUMB *sequence.

The identification of an additional potentially spliced exon within the *NUMB *gene raises the possibility that six and up to eight distinct human splice variants may exist. To determine if this was the case, western blotting of cellular lysates was conducted on NT2 and human MDA-MB-231 cells where the potential extra numb amplicons were readily detected by PCR. To ensure these novel protein bands corresponded to NUMB, the western blot was probed with the carboxy-terminal numb antibody and all bands were immune reactive (Figure 1C). To truly test for its validity, the bands corresponding to the correct predicted molecular weights were excised from the Coomassie blue stained gel and subjected to mass spectrometry analysis. The peptide fragments isolated and characterized are shown in Additional file 2. In both cases the peptide fragments confirm that the bands in Figure 1D are in fact NUMB, confirming the existence of at least two additional *NUMB *splice variants in which exon 10 was spliced out of the mature cDNA. To be consistent with the previously reported NUMB nomenclature [5], we name these novel isoforms NUMB5 (PTB^LONG^exon10^-^PRR^SHORT^, ABY89090.1) and NUMB6 (PTB^SHORT^exon10^-^PRR^SHORT^, ABY89091.1) (Figure 1A).

### NUMB as a potential oncogenic protein

**Figure 2:**
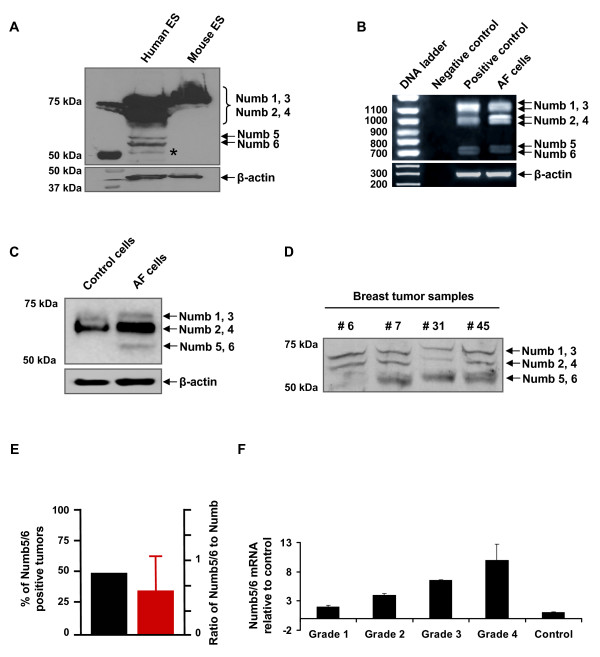
**Numb5 and Numb6 expression**. **(A) **Western blot detection of Numb5 and Numb6 isoforms from a Numb immunoprecipitation of human and mouse embryonic stem (ES) cells. Another, unknown Numb isoform is indicated with an asterisk. This is confirmed to be Numb from our mass spectrometry analysis and protein mobility on the gel. **(B) **mRNA analysis of *NUMB *isoforms in human amniotic fluid (AF) cells by RT-PCR; the AF cells served as a surrogate for human ES cells. Negative control, no template; positive control, NT2/D1 cells after 4 days of retinoic acid treatment; β-actin served as control for the RNA input. **(C) **Western blot analysis of NUMB5 and NUMB6 in AF cells. Control, the postnatal mouse cortex; loading control, β-actin. **(D) **Western blot detection of NUMB5 and NUMB6 in breast tumor samples. **(E) **Real-time PCR analysis of the mRNA extracted from 50 individual mammary tumor samples. The left y-axis and black bar depict the percentage of *NUMB5*- and *NUMB6*-positive tumors using the primers specific for each splice variant. The right y-axis and red bar show the ratio of *NUMB5 *and *NUMB6 *mRNA to total *NUMB *mRNA within each sample. **(F) **Real-time PCR analyses of the *NUMB5 *and *NUMB6 *mRNAs extracted from breast tumor specimens in different grades of tumor grades, showing Numb5/6 expression linearly correlated with tumor progression. NT2 cells served as control. Tumor grading was based on Bloom-Richardson system, Error bars indicted standard error of the mean.

In order to understand the genesis of NUMB5/6 expression, we conducted Western blot analysis of naive human embryonic stem cells to confirm expression of NUMB5/6 in early development (Figure 2A). We also obtained amniotic fluid cells from routine amniocentesis of pregnant women ranging from 15 to 35 weeks of gestation and cultured the cells as described previously [28] for mRNA (Figure 2B) and protein analysis (Figure 2C).

Using the same PCR and Western blot approach that identified NUMB5 and NUMB6, we examined de-identified breast tumor biopsy samples for the expression of *NUMB5/6 *mRNA. The larger specimens were disaggregated and subjected to Western blotting (Figure 2D). Since *NUMB *is not expressed in normal breast tissue, it was surprising that over 50% of the tumors examined (26 of 50) expressed *NUMB5/6 *mRNA (Figure 2E, black bar). These data were further verified by real-time PCR, showing that *NUMB5/6 *transcripts comprise approximately 60% of the total *NUMB *transcripts in these samples (Figure 2E, red bar, scale on right ordinate). Furthermore, the expression level of *NUMB5/6 *correlated with the clinical grade (pathology performed blind to the investigators) of the specimen (Figure 2F).

### Numb5/6 expression *in vivo *leads to tumor formation

**Figure 3:**
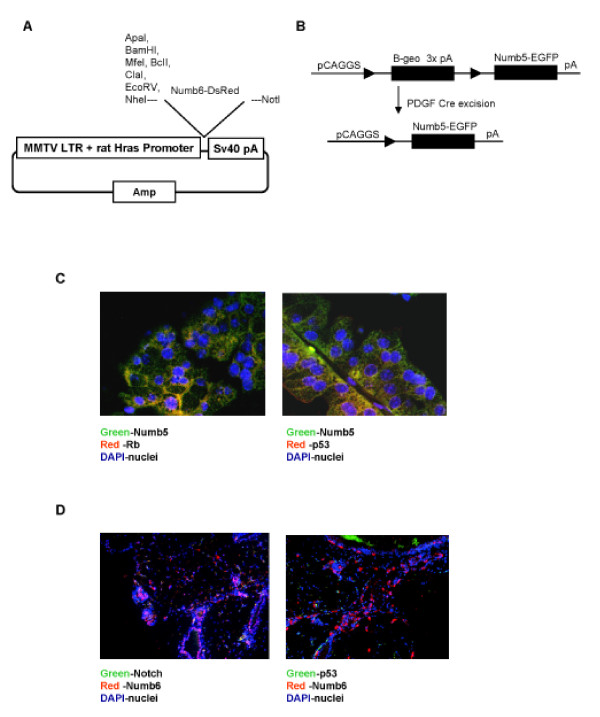
**Numb5 and Numb6 expression in tumor development**. **(A) **Schematic of the over-expression plasmid used for MMTV-NUMB6-DsRed transgenic mouse production. **(B) **Schematic of the Cre recombinase over-expression plasmid used for NUMB5-EGFP transgenic mouse production. **(C) **Immunohistochemistry analysis of retinoblastoma (Rb) and p53 in the brain and retinal tumors. Green indicates Numb5 transgenic over-expression, and red Rb or p53 antibody detection; yellow shows co-localization of the two proteins. Blue visualizes Dapi staining of the nucleus. **(D) **Immunohistochemistry analysis of the Notch intracellular domain and p53 in the retinal tumor. Red shows Numb6 expression in tissue derived from MMTV transgenic mouse. Green shows Notch or p53 Immunofluorescence detection. Yellow indicates co-localization of the two proteins. Blue is Dapi staining of the nucleus. LTR, long-terminal repeat; PDGF, platelet-derived growth factor.

To begin addressing a causal role for Numb5/6 in tumorigenesis, we created two sets of transgenic mice, one in which Numb6 expression is driven by the mouse mammary tumor virus (MMTV) promoter and another where Numb5 is conditionally expressed by the tissue-specific expression of Cre recombinase (Figure 3A-B). It was our expectation to create novel cancer models to better understand the mechanisms of Notch and Numb regulation in cancer. Both transgenic mouse models led to tumor formation, MMTV-Numb6-DsRed in the breast and platelet-derived growth factor (PDGF)-Cre-Numb5 in the retina and cortex with 25% penetrance. Immunohistochemical staining of these overgrowths revealed that retinoblastoma (RB) and p53 protein expression was induced in the retinal tumors (the PDGF-Cre-Numb5 derived overgrowth was 2.5 times the size of the eye itself). We found that the Notch intracellular domain (NICD) and p53 were not significantly up regulated in the MMTV-Numb6 derived breast overgrowth (Figure 3D). Despite the low penetrance of these overgrowths, it appears to be clear that Numb5/6 has a potential role in tumor formation, and may not be a signaling panacea to fight tumor formations in aberrant Notch-signaling-induced overgrowths.

### The effect of NUMB5 and NUMB6 on Notch

**Figure 4:**
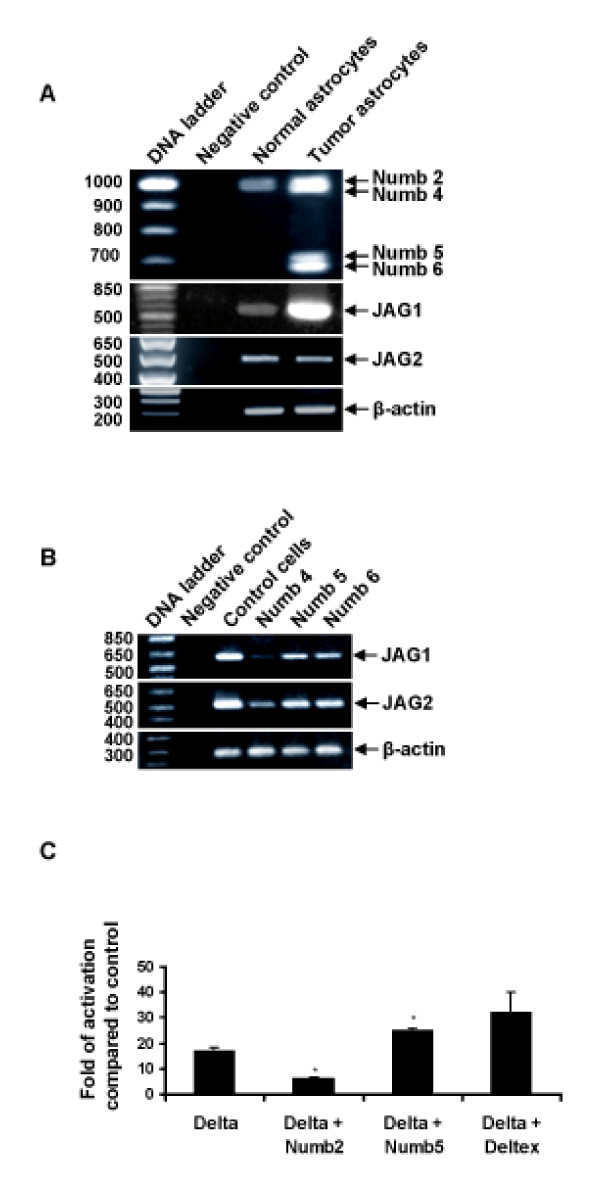
**NUMB5 and NUMB6 expression effects Notch signaling**. **(A) **RT-PCR analysis of mRNA extracted from brain glioblastoma U87 cells. Negative control, no template; loading control, β-actin. **(B) **RT-PCR analysis of *JAG1 *and *JAG2 *in the HEK293GPG cells infected with NUMB4, NUMB5 or NUMB6. Negative control, no template; control, no infection; loading control, β-actin. **(C) **Luciferase assay of Notch activation by Delta in N2a cells expressing NUMB isoforms using CSL-luciferase vector. Data were obtained from five independent experiments (in quadruplicate), and are presented as mean + standard deviation. *P < 0.025.

The current notion that activated Notch signaling can lead to tumor formation provided the rationale to examine the levels of NUMB5/6 and known activators of Notch signaling in the glioblastoma cell line U87 and to compare them to the levels found in cultured human astrocytes. Interestingly, the expression of NUMB6 coincided with the up-regulation of the Notch ligand Jagged (JAG)1 in glioblastoma cells (Figure 4A), raising the possibility that NUMB5/6 may influence Notch differently to the other NUMB isoforms. To examine this possibility, HEK293 cells were transfected with NUMB4, NUMB5, OR NUMB6 and examined for JAG1 and JAG2 expression by RT-PCR (Figure 4B). Comparative analyses show that NUMB4 dramatically down-regulated the expression of JAG1 (80%) and JAG2 (55%). In contrast, NUMB5 and NUMB6 had little effect on expression of JAG1 (15% reduction in each case) and JAG2 (10% reduction in each case). Furthermore, using two distinct luciferase reporter constructs (Hes-1 luciferase [29] and CSL-luciferase [30]), NUMB5 (Figure 4C) and NUMB6 (data not shown) had no negative effect on luciferase production, whereas NUMB2 over-expression reduced the Hes-1 and CSL-luciferase signal, indicating that NUMB2 inhibits Notch signal transduction but NUMB5/6 do not.

### The effect of NUMB5 and NUMB6 on cytoskeletal organization and migration

**Figure 5:**
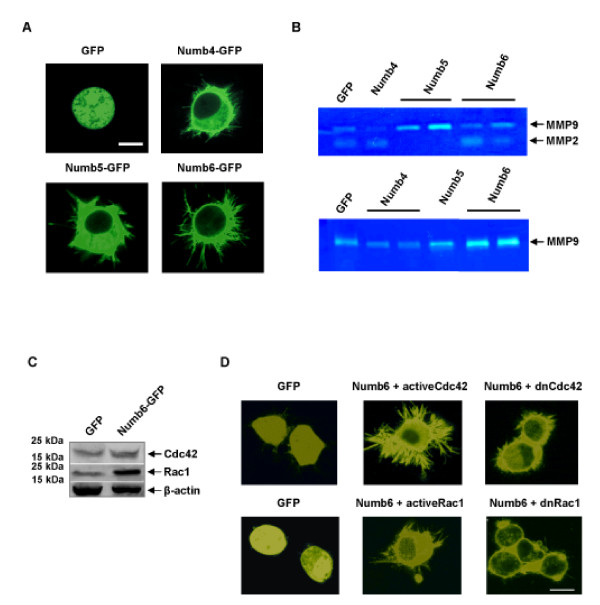
**Numb5 and Numb6 enhance lamellipodial and filopodial growth, respectively**. **(A) **Morphological analyses of cytoplasmic protrusion formation in N2a cells expressing Numb5-GFP and Numb6-GFP. **(B) **Zymographic detection of MMP2 and MMP9 gelatinases secreted to conditioned media in DB7 (upper panel) and Met-1 (lower panel) cells over-expressing GFP, Numb4, Numb5, and Numb6. **(C) **Western blot analyses of Cdc42 and Rac1 protein expression in N2a cells over-expressing Numb6-GFP. **(D) **Confocal immunofluoresence analysis of filopodial growth regulation by Cdc42 (upper panel) and Rac1 (lower panel) GTPases in N2a cells over-expressing Numb6-GFP. The Numb6 induced filopodial growth is significantly abrogated by dominant negative (dn) Cdc42 and dominant negative (dn) Rac1. Scale bar = 10 μm.

In addition to their effects on Notch signaling, another important feature that distinguishes Numb5/6 from the other Numb isoforms is the production of membrane protrusions. There is accumulating evidence that lamellipodia and filopodia are directly involved in the formation of growth cones and differentiation of progenitors into neurons [31]. Ectopic expression of Numb5 and Numb6 induced the rapid formation of lamellipodia (Numb5) and filopodia (Numb6) in. cortical progenitors (Additional file 3A) and N2a cells (Figure 5A). The number of protrusions is higher in Numb5/6 overexpressing cells as compared to green fluorescent protein (GFP) control in both cell lines (Additional file 3B). Furthermore, unlike growth cones, the Numb5- and Numb6-induced lamellipodia and filopodia were random and did not appear to follow any directional cues (Additional file 3A). To gain further information on their potential role in tumor metastases, the expression of Numb5/6 d id lead to increases in matrix metalloproteinase MMP2 and MMP9 secretion compared to the expression of Numb4 as shown by zymographic analysis of cells stably expressing these Numb isoforms (Figure 5B).

It has been convincingly demonstrated that the Rho GTPases, particularly Cdc42 and Rac1, play important roles in the formation of lamellipodia and filopodia through regulating the actin cytoskeleton [32-35]. Although both Numb5 and Numb6 gave similar induction results, we show Numb6 as the representative isoform for the following experiments for its higher number of protrusions. We observed up-regulation of Cdc42 and Rac1 upon over-expression of Numb6 compared to the GFP control (Figure 5C). We performed immunostaining and over-expression studies using Cdc42 and Rac1 to further address these questions. Confocal microscopy confirmed that Numb6 and Cdc42 are co-localized in the filopodial protrusions of cortical progenitors (Additional file 3C,D) and N2a cells (Figure 5D) as early as 24 hours after Numb6 expression. In addition, the co-expression of Numb6 and the constitutively active Cdc42 led to an increase in filopodial extensions in N2A cells (Figure 5D), suggesting that Cdc42 may regulate Numb6-induced cytoskeletal changes. To ensure that Cdc42 is directly involved in this process, we also tested the effect of dominant negative Cdc42 (Cdc42N17), establishing that filopodial extensions are abrogated in the presence of an inhibitory Cdc42 protein (Figure 5D). Further examination showed that the co-expression of Numb6 with Rac1 enhanced filopodial extensions, whereas dominant negative Rac1 had a negative effect on filopodia formation (Figure 5D). Moreover, the same protein was able to ameliorate lamellipodia extenstions in Numb5-expressing cells. Together, these results demonstrated that the induction of filopodial growth by Numb6 and lamellipodia extensions via Numb5 is regulated by Cdc42 and Rac1.

### The interaction of Numb5 with polarity-regulating proteins

**Figure 6:**
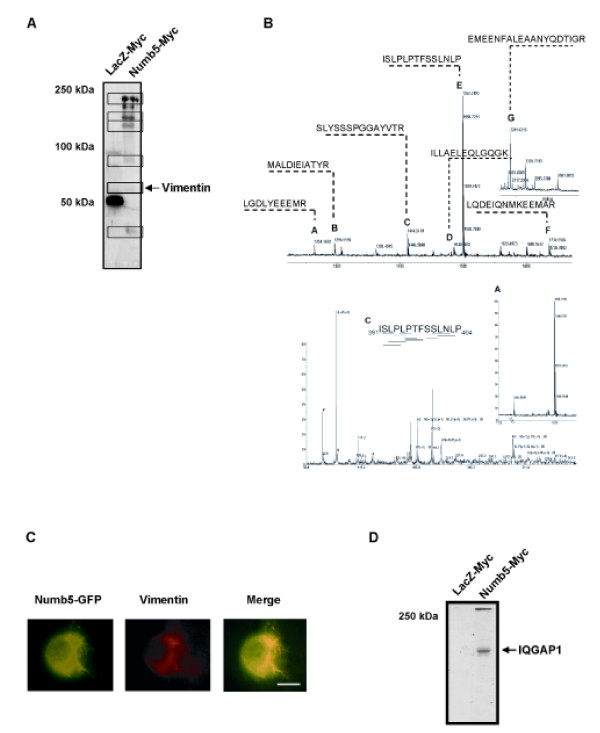
**Numb5 interacts with cytoskeleton and polarity regulating proteins**. **(A) **Immunoprecipitation assay of cellular proteins interacting with Numb5 using Myc antibody. Lanes represent protein separation in N2a cells expressing LacZ-Myc-His and Numb5-Myc-His. The potential interacting proteins are shown in boxes. **(B) **Mass spectral sequence analysis of the protein band at approximately 53 kDa. Peptide mapping analysis confirms that the Numb5-interacting protein is vimentin (upper panel). The sequences of peptide fragments generated for vimentin residues from amino acids 391 to 404 (lower panel). **(C) **Confocal immunofluorescence detection of vimentin protein in N2a cells over-expressing Numb5-GFP. Green shows Numb5 detection in N2a cells infected with Numb5-GFP; red shows vimentin protein expression detected with vimentin antibody and Alexa546 fluorophore; yellow shows the merged image. Scale bar = 7 μm. **(D) **Western blot detection of IQGAP1 protein in N2a cells over-expressing Numb5-Myc. Numb5 interacts with the polarity and migration regulating protein IQGAP1. Total protein lysates from N2a cells expressing LacZ-Myc-His or Numb5-Myc-His were electrophoretically separated and subjected to Western blotting using IQPAP1 antibody.

To identify proteins that interact with Numb5 (Numb5 immunocomplexes generated the most reliable ions in mass spectrometry analyses compared to Numb6), LacZ-Myc-His and Numb5-Myc-His expressing N2a cell lysates were immunoprecipitated with Myc antibody and subjected to SDS-PAGE (Figure 6A). The bands identified as potential co-immunoprecipitated candidates were removed, digested and analyzed using a QSTAR mass spectrometer. The bulk number of the peptides was mapped to Cdc42, atypical protein kinase C (aPKC), vimentin, and IQGAP (IQ (motif) GTPase activating protein 1). Among them, Cdc42 and aPKC were previously shown to interact with Numb [36,37]. Thus, we focused our studies on two putative interacting proteins, vimentin and IQGAP, that are known to be involved in cytoskeletal arrangement. We selected vimentin (Figure 6B) for further protein sequence analysis. Using immunocytochemistry, we show that vimentin is co-localized with Numb5 over-expression in the cytoplasm and the membrane protrusions of N2a cells (Figure 6C). Similar results were obtained using Numb6 and examining lamellipodia protrusions (data not shown). To confirm IQGAP interaction with Numb5, we carried out western blot analyses with the LacZ-Myc-His and Numb5-Myc-His expressing N2a cell lysates, showing that Numb5 does interact with IQGAP1 (Figure 6D).

### The effect of Numb5 on cell migration

**Figure 7:**
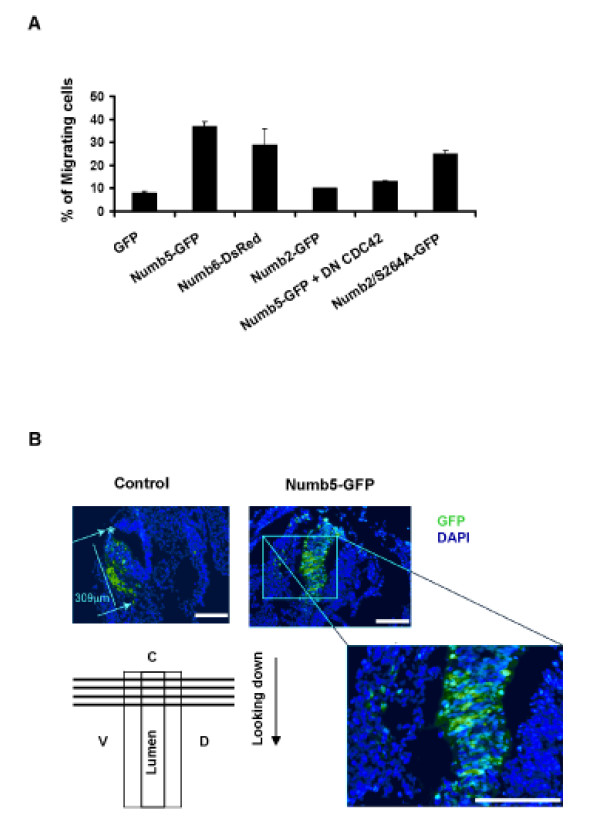
**Ectopic Numb5 and Numb6 expression alters cell migration *in vitro *and *in vivo***. **(A) **Migration of neural crest cells expressing Numb5-GFP or Numb6-DsRed *in vitro *at 24 hours using the Boyden chamber. NUMB5-GFP or NUMB6-DsRed expressing neural crest cells demonstrate significantly higher migration than their GFP or NUMB2-GFP expressing counterparts. Dominant negative (dn) CDC42 and, to a lesser degree, NUMB2 mutation (NUMB2/S264A) tend to retain normal migration. Data are presented as means ± standard deviation using three experiments originating from discrete infections. **(B) ***In vivo *migration analysis of neural crest cells expressing Numb5-GFP eletroporated into stage 4 chick embryos. Numb5-GFP over-expression leads to irregular abrogated neural crest migration in the chick embryos. The confocal images of stage 4 chick embryos were taken 12 hours after GFP or Numb5-GFP electroporation into the dorsal region. Green shows GFP expression; DAPI indicates nuclei staining.

Knowing that Numb has antagonistic effects on Notch, and that Notch is known to induce cell migration, we examined the ability of Numb5/6 to regulate cell migration using the classic Boyden chamber assay. Indeed, when compared to Numb4, Numb5/6-expressing cells demonstrated a significant increase in cell migration in vitro (Figure 7A). Numb5 and Numb6 are devoid of serine 264, a serine residue that is present in other Numb isoforms and is critical in the formation of complexes with 14-3-3 proteins. Therefore, we tested whether NUMB2 mutant carrying alanine (A) instead of serine (S) 264 (NUMB2/S264A) may affect cell migration. When this mutant was expressed in N2a cells, migration was increased; a phenomenon further enhanced in the Numb5 and Numb6 over-expressing cell lines (Figure 7A). Co-transfecting dn-CDC42 along with the Numb5-GFP construct inhibited the cell migratory affects of the Numb5 alone. These results showed that the absence of PRR1 (see Figure 1), particularly serine 264, is involved in the NUMB5- and NUMB6-induced cell migration.

In order to test whether Numb5 could also change the migratory behavior of cells *in vivo*, Numb5-GFP was electroporated into the surface of stage 4 chick neural tube. Following electroporation, eggs were incubated for 12 hours to allow neural crest cells to commence migration. The dorsal trunk neural tube electroporated with control GFP, (Figure 7B, left) or Numb2-GFP (data not shown) showed normal neural crest migration. In contrast, the embryos electroporated with Numb5-GFP demarcated cell migration back into the tube and not in the classic dorsal-peripheral migration stream (Figure 7B, right). This aberration in migratory pattern was more evident with Numb5-GFP cells migrating only 80 μm from where the electrode and DNA were placed, compared to 309 μm for control GFP cells. Together, these data show that Numb5 has the capacity to change cell migration *in vivo*.

## Discussion

This study, for the first time, provides evidence for the existence of two and up to four human NUMB novel splice variants, NUMB5 and NUMB6. These isoforms differ from the known NUMB variants (NUMB1-4) in structure and function. NUMB5 and NUMB6 lack PRR1 and interact with CDC42, vimentin and IQGAP1. In addition, they induce lamellipodial and filopodial growth (under the regulation of CDC42 and RAC1). The structure function of NUMB5/6 allows for potential docking of key mediators in cancer malignancies. It is well know that both CDC42 and RACI influence cell growth and migration. NUMB5/6 promote the initial stages of neural protrusions required for cellular migration.

Several reports in the past few years have shown that Numb is involved in the polarity of neural progenitors [38] and neurons [5,24,30,36,39]. However, information on the specific Numb isoforms that are capable of such changes has been lacking. It remains to be elucidated how the structural differences among Numb isoforms contribute to their functional differences in cell polarity. The recent examination of Numb in dendritic spines suggests that structural changes in Numb (PRR deletion) or its mutation can lead to lamellipodial and filopodial growth [40,41]. One possibility is that the deletion of Numb PRR1 may allow for more efficient interaction between Numb and proteins such as Cdc42 and Rac1, which are involved in the induction of membrane protrusions. The expression of constitutively active Cdc42 has been shown to induce both filopodia and the Rac1-dependent lamellipodia [40,41]. Our data show that the Numb6-induced filopodial growth occurs as a direct result of Cdc42 interaction and regulation. However, none of the large number of processes formed developed into a mature axon. A detailed examination of the Cdc42 signaling cascade confirms that although the initial events in actin reorganization are required for neurite outgrowth, this process is not completed without the proper assembly of microtubules [42]. While actin and microtubule cytoskeletons are re-organized during cell polarization, the actin crosslinking protein IQGAP1 facilitates cell migration [43]. To perform this function, IQGAP1 binds to Cdc42 and Rac1, maintaining Cdc42 in an active state, and contributes to local actin assembly at the leading edge of migrating cells [43]. In our experiments, Numb5 and Numb6 showed interactions with Cdc42, IQGAP1 and vimentin, a protein expressed in radial glial and glioblastoma cells. The co-localization of Numb and vimentin in lamellipodia and filopodia (this study) and the apical end-feet of radial glial cells [38] further support the role of Numb in cell polarity during development. This function is also accompanied by Numb-mediated regulation of Notch signaling to maintain cell homeostasis [16,17,44]. Our data show, for the first time, that Numb isoforms have different antagonistic effects on Notch signaling. The high levels of JAG1 expression in high grade glioma and glioblastoma cells (this study; also see [18]) can be significantly down-regulated by Numb2 and Numb4 but not by Numb5 and Numb6. Since the regulation of Notch signaling by Numb is considered as a method to treat breast cancers [19,44], future examination and utilization of Numb isoforms can contribute to the development of proper treatment for glioma and glioblastoma. The same is true for breast tumors in which the Numb5/6 status correlates with clinical and pathological parameters of aggressive neoplasms [16,19]. Therefore, it is reasonable to suggest that Numb isoforms have the capacity to serve as biomarkers for prognosis and as potential therapies for cancer.

## Materials and methods

### Cell culture

Cortical progenitors and astrocytes were cultured as previously described [26,27,45]. In brief, neural progenitors were obtained from embryonic day 13 (E13) mouse telencephalon and grown in DMEM, high glucose, L-glutamine (Invitrogen, San Diego, CA, USA) plus 5% fetal bovine serum (ThermoScientific, Hyclone. Rockford, Il, USA) plus N2 Supplement (Invitrogen). Parallel cultures were used for neuronal differentiation by using 0.5% fetal bovine serum and 1 μM cytosine arabinoside (Sigma-Aldrich, St. Louis, MO, USA) during the course of experiments. Amniotic fluid cells were obtained with consent from pregnant woman undergoing amniocentesis at Ottawa Hospital (Ottawa, Canada) and following all practices of the National Research Council and Ottawa General Hospital in accordance with all rules and guidelines of both the Ottawa Hospital and National Research Council of Canada. The mouse mammary carcinoma low metastatic DB-7 and high metastatic Met-1 cell lines were provided by D Spicer (Maine Medical Center Research Institute, Scarborough, ME, USA). For the generation of stable clonal lines expressing control GFP and Numb4, Numb5 and Numb6 CMV-GFP constructs, selection was carried out in medium supplemented with 0.5 mg/ml of G418 sulfate (Calbiochem, Gibbstown, NJ, USA). After selection cell were maintained with 0.2 mg/ml of G418.

### RT-PCR and quantitative PCR

RNA was extracted using a micromRNA isolation kit (Agilent Technologies, Stratagene, Santa Clara, CA, USA) and then treated with RNase-free DNase and synthesized into cDNA [46,47]. NUMB (NUMB1, 1,159 bp; NUMB2, 1,015 bp; NUMB3, 1,126 bp; NUMB4, 982 bp; NUMB5, 688 bp; NUMB6, 721 bp), JAG1 (558 bp), JAG2 (485 bp), and ACTIN B (β-ACTIN (ACTB), 294 bp) cDNA fragments were amplified using the following conditions: NUMB (40 cycles): 94°C (3 minutes), 94°C (1 minute), 55°C (30 s), 72°C (1 minute), 72°C (5 minutes) (NUMB-F, AGGAATGCACATCTGTGAAG; NUMB-R, CTCAGAGGGAGTACGTCTAT). JAG1 (28 cycles), JAG2 (28 cycles) and ACTB (25 cycles): 95°C (5 minutes), 95°C (1 minute), 60°C (1 minute), 72°C (1 minute), 72°C (10 minutes) (JAG1-F, ACACACCTGAAGGGGTGCGGTATA; JAG1-R, AGGGCTGCAGTCATTGGTATTCTGA; JAG2-F, CAGTGGCTTTACTGGCACCTACTGC; JAG2-R, GGGTTGCAGTCGTTGGTATTGTGAG; ACTB-F, TCACCCACACTGTGCCCATCTACGA; ACTB-R, CAGCGGAACCGCTCATTGCCAATGG). The resulting amplicons were separated on a 1 to 1.5% TAE gel including 0.5 μg/ml ethidium bromide, cloned and sequenced.

Quantitative PCR was performed according to the Livak method of quantification [48]. In brief, Numb5 and Numb6 primers were used to create approximately 90-bp amplicons with a primer within the PTB domain and common to all Numb isoforms and a primer that annealed to the junction of exons 9 and 11. In a separate reaction, the endogenous Numb was examined using the same PTB domain primer and a primer 20 bases beyond the putative exon 9 and 11 splice site.

### Luciferase assays

N2a cells were transiently transfected with Hes-1- or CSL-dependent luciferase constructs, the herpes thymidine kinase-driven *Renilla *luciferase (Promega, Madison, WI, USA) as a control for transfection efficiency and Lipofectamine 2000 (Invitrogen) in accordance with the manufacturers' instructions. Each well contained 100 ng of Notch reporter plasmid, 2 ng of the *Renilla *luciferase plasmid and 200 ng of either the LacZ-Myc-His construct or the relevant Numb Myc-His. Dual luciferase assays (Promega) were performed in quadruplicate, using 24-well tissue culture plates (ThermoScientific. Nunc products) 24 hours after the initiation of transfection.

### Zymographic analysis of MMP activity

DB-7 and Met-1 cell cultures of 70 to 80% confluence over-expressing GFP, Numb4, Numb5, and Numb6 were washed twice with PBS, and the medium was changed to serum free DMEM-F12 without supplements. After 48 hours, the conditioned medium was collected and centrifuged for 5 minutes at 400 × g. A 500-μl aliquot was concentrated to <100 μl in a Microcon concentrator (Millipore, Billerica, MA, USA) at 6,500 × g at 4°C. Protein concentration was determined using a BCA assay (Thermo Scientific, Rockford, IL, USA), and 5 μg of total protein from each sample was electrophoresed on a 10% zymography gel containing 0.1% gelatin (Invitrogen). MMP activity was detected by incubating the gel in 1× zymogram renaturing buffer for 30 minutes at room temperature and then in 1× zymogram developing buffer (Invitrogen) overnight at 37°C, followed by staining with SimplyBlue stain (Invitrogen). Staining gels were air-dried in cellophane mounts and images were then captured.

### Retroviral gene delivery

HEK 293GPG cells were transfected with AP2-IRES-EGFP, AP2-NUMB-EGFP or AP2-NUMB-DsRed vectors, with NUMB representing a specific isoform. Each NUMB construct carried only a specific NUMB isoform, depending on the experiment. The retroviral particles were collected every 24 hours, titrated and applied to target primary cultures three times over 48 hours.

### Antibodies

Antibodies to the following were used in this study: Numb (kindly provided by Dr Kozo Kaibuchi; also Millipore, Upstate, Billerica, MA, USA), IQGAP1 (MBL International, Woburn MA, USA), CDC42 (Cell Signaling Technology, Danvers, MA, USA), phospho-Rac1 (Cell Signaling), GFAP (Neomarkers, Fremont CA, USA), Myc (Santa Cruz Biotechnology Santa Cruz. CA, USA; Cell Signaling), β-actin (Sigma-Aldrich), Alexa Fluor 488-conjugated IgG (Invitrogen Molecular Probes), rhodamine-conjugated IgG (Jackson Laboratories, Bar Harbor, ME, USA) and horseradish peroxidase (Jackson). F-Actin was detected by incubation with phalloidin (Molecular Probes).

### Immunoblotting and immunofluorescence

Cells were lysed in NP-40 buffer (1% NP-40, 0.1% SDS, 0.15 M NaCl, pH 7.2), cleared by centrifugation, and applied to western blots [27]) or immunoprecipitation. In the immunofluorescence experiments, cells were rinsed with PBS, fixed with 70% ethanol plus 0.15 NaCl (10 minutes), and treated with 10% goat serum plus 0.05% Triton X-100 for 30 minutes. Cells were washed with PBS and primary antibodies applied, then washed with PBS again and the secondary antibodies applied. Images were taken using a Axiovert 200 microscope (Zeiss) and a laser-scanning confocal microscope (LSM, Leica) and processed using Adobe Photoshop.

### In-gel digestion

In-gel digestion was performed using a modified version of a well-established method [49]. Protein bands (0.3 g) were excised and minced using a new razor blade, and the pieces were de-stained, dried in a SpeedVac and rehydrated (25 mM NH_4_HCO_3_, pH 8.0 and 0.01 g/ml trypsin). The pieces were overlaid with 25 mM NH_4_HCO_3 _and incubated for 15 hours at 37°C. Peptides were recovered by three extractions of the digestion mixture with 50% Acn-5% trifluoroacetic acid. All supernatants were pooled and concentrated in the SpeedVac. The peptide mix was stored at 20°C until analysis.

### MALDI-TOF mass spectrometry of Numb-binding proteins

Peptide aliquots of un-separated tryptic digests were co-crystallized with cyano-4-hydroxycinnamic acid and analyzed using a matrix-assisted laser desorption ionization (MALDI) delayed-extraction reflectron time of flight (TOF) instrument (Voyager Elite mass spectrometer; Applied Biosystems, Carlsbad, CA, USA) equipped with a nitrogen laser. Measurements were performed in a positive ionization mode. All MALDI spectra were externally calibrated using a standard peptide mixture. Some post source decay spectra were acquired on a TOF Spec SE MALDI-TOF mass spectrometer (Micromass Limited, Beverly, MA, USA) with a nitrogen laser and operated in the reflectron mode.

### Numb5/6 protein sequence analysis by MS/

The protein extracts from NT2 and N2a cells were immunoprecipitated with anti-Numb antibody specific for conserved 600 amino acid stretches at the carboxyl terminus of all Numb isoforms (Abcam, Cambridge, MA, USA). Numb immunocomplexes were washed with lysis buffer and separated by polyacrylamide SDS-PAGE. Two bands with molecular weights of 54 and 55 kDa from each NT2 and N2a cells were excised from the gel for protein mapping and mass spectrometry sequence analyses. For the electrospray analysis, samples were reduced, alkylated, and digested with trypsin (sequencing grade; Promega) following a standard protocol. A sample was applied to an RP-18 precolumn (LC Packings, Amsterdam, The Netherlands) using the 0.1% trifluoroacetic acid mobile phase and then transferred to a nano-HPLC RP-18 column (LC Packings) using a linear acetonitrile gradient of 0 to 45% acetonitrile in water over 30 minutes in the presence of 0.05% formic acid at a flow rate of 40 nL/minute. The column outlet was directly coupled to the nano-Z-spray ion source of the Q-Tof electrospray mass spectrometer working in the regime of data dependent MS to MS/MS switch, allowing for a 3 s sequencing scan for each detected peptide. Spectra were internally calibrated using trypsin autoproteolysis peaks, and the accuracy of mass measurements of all peptides was in the range of ±0.05 Da. The β-actin band was analyzed as a positive control for both peptide mapping and protein sequencing. The data were analyzed using Analyst QS (Agilent Technologies, Santa Clara, CA, USA).

### *In ovo *neural tube electroporation

Fertilized white leghorn chicken eggs were incubated until Hamburger-Hamilton stage 4 at 37°C in a humidified incubator. The eggs were windowed and injected with a solution of 3% India ink in Ringer's solution below the blastoderm to enhance the contrast. The vitelline membrane overlying the embryo was removed and the plasmid DNA (CAXGFP, the combined cytomegalovirus immediate early enhancer CMV IE and chicken beta-actin promoter driving EGFP, NUMB5-GFP or NUMB6-DsRed) was injected into the dorsal neural tube. Approximately 0.5 ml of Ringer's solution was placed on top of the embryo. The anode (positive electrode) was placed such that the negatively charged DNA was pulled into the desired portion of the neural tube. The electrodes consisted of two 1-mm platinum wires connected to a square pulse electroporater (BTX, San Diego, CA, USA) set to deliver five 50-ms pulses of 9 to 15 V.

After electroporation, eggs were covered and returned to the incubator. After 24 hours, embryos were removed from the egg and fixed in 4% paraformaldehyde at 4°C overnight. After washing in PBS, embryos were embedded in 7.5% low melting agarose and sectioned at 100 μm by a vibratome. Sections were incubated with enhanced GFP (EGFP; Molecular Probes) antibody at 4°C overnight, washed in PBS and incubated with the secondary antibody (Alexa 488; Molecular Probes) at room temperature for 1 hour.

### Transgenic mouse production and tumor excision

Numb6-DsRed was ligated into the MMTV-pA vector via the NheI and NotI sites then purified for microinjection into FVB/N mouse embryos. Numb5-EGFP was ligated into the Z/EG vector construct via the BGLII and NotI sites, then purified for microinjection into FVB/N mouse embryos. Tumors were excised from euthanized mice and placed in 4% paraformaldehyde overnight for the paraffin to set and then sectioned. Slides were washed in xylene for 10 minutes each then three series of decreasing ethanol washes. Immunofluorescence experiments were then performed.

## Abbreviations

aPKC: atypical protein kinase C; bp: base pair; DMEM: Dulbecco's modified Eagle's medium; EGFP: enhanced green fluorescent protein; GFP: green fluorescent protein; IQGAP1: IQ (motif) GTPase activating protein 1; JAG: Jagged; MALDI: matrix-assisted laser desorption ionization; MMP: matrix metalloproteinase; MMTV: mouse mammary tumor virus; PBS: phosphate-buffered saline; PTB: phosphotyrosine binding; PRR: proline rich region; TOF: time of flight.

## Competing interests

The authors declare that they have no competing interests.

## Authors' contributions

AK carried out the zymographic analyses, immunoprecipitations, Numb5/6 protein sequencing and the majority of the Western blotting. MBY performed the initial PCRs involving Numb5/6. CK contributed to the initial cloning and characterization of Numb5/6 as well as participated in the sequence alignment. CV preformed all the MALDI-MS/MS analysis. DS preformed all the neural crest migration experiments. JMV conceived the design of the study, and was responsible for its coordination. TLA served as chief technical assistant in all experiments. All authors read, collaborated and helped in drafting the manuscript.

## Supplementary Material

Additional file 1 **Supplemental Figure 1**. Human NUMB isoform detection using PCR. **(A) **Schematic of the PCR strategy utilized for both original cloning and further characterization of NUMB5 and NUMB6. **(B) **Genomic architecture of the NUMB splice variants. PRR, proline rich region; PTB, phosphotyrosine binding domain. **(C) **RT-PCR shows NUMB5 and NUMB6 expression throughout NT2/D1 cell differentiation time points using retinoic acid treatment. Negative control, no template; loading control, β-ACTIN at 100 bp. Click here for file

Additional file 2 **Supplemental Table 1**. Peptides found in tryptic digests of Numb isoform 5 and 6 electrophoretic bands as indicated in Figure 1D. Click here for file

Additional file 3 **Supplemental Figure 3**. NUMB5 and NUMB6 enhance lamellipodial and filopodial growth. **(A) **NUMB5 and NUMB6 over-expression in neural progenitors enhances lamellipodial and filopodial extension, respectively. Arrowheads indicate lamellipodia; white arrowheads indicate filopodia. **(B) **Corresponding quantification of protrusion number in GFP, NUMB5, and NUMB6 overexpressing cells. **(C) **Confocal imaging shows that NUMB6 co-localizes with CDC42 in neural progenitors. NUMB6-DsRed infection, green CDC42 antibody staining, yellow, both merged. White arrowheads indicate filopodia. Scale bar = 5 μm. **(D) **Using confocal imaging, NUMB6 is co-localized with CDC42 in N2a cells. NUMB6-DsRed infection, green CDC42 antibody staining, yellow, both merged. Click here for file
