# Supplementary figures and images for: Two novel human NUMB isoforms provide a potential link between development and cancer
Source: Neural Dev. 2010 Dec 1;5:31. doi: 10.1186/1749-8104-5-31 (PMC3009962; doi:10.1186/1749-8104-5-31)

Supplementary Figure 1

A

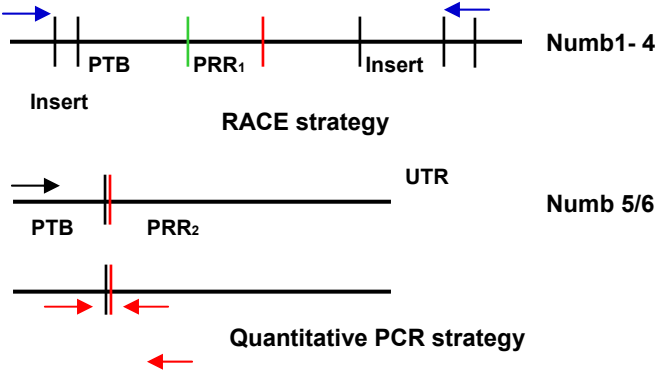

B

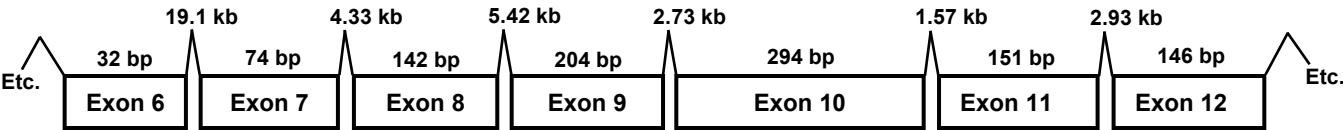

C

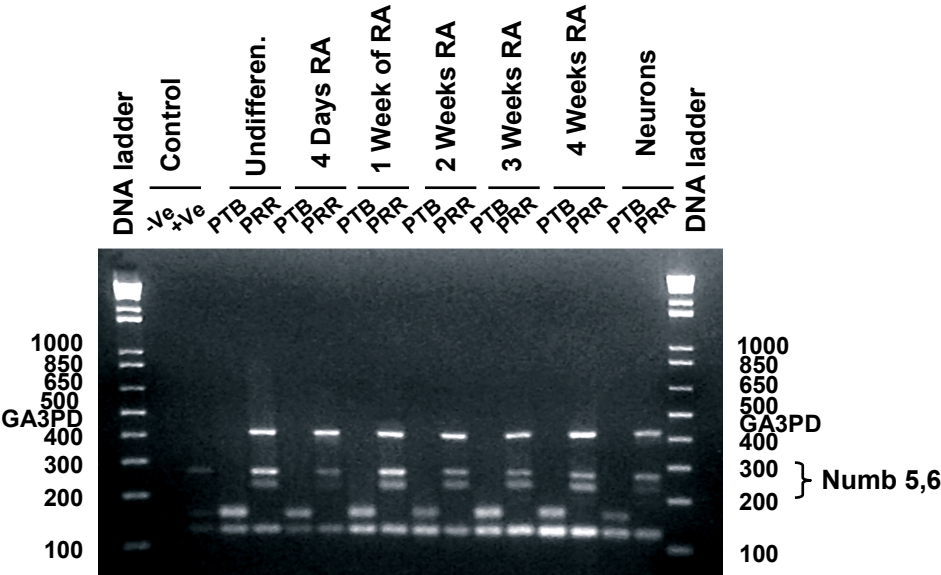

Supplement: Additional file 1 — Supplemental Figure 1. Human NUMB isoform detection using PCR. (A) Schematic of the PCR strategy utilized for both original cloning and further characterization of NUMB5 and NUMB6. (B) Genomic architecture of the NUMB splice variants. PRR, proline rich region; PTB, phosphotyrosine binding domain. (C) RT-PCR shows NUMB5 and NUMB6 expression throughout NT2/D1 cell differentiation time points using retinoic acid treatment. Negative control, no template; loading control, β-ACTIN at 100 bp. [file 1749-8104-5-31-S1.PDF]

Supplementary Figure 3

A

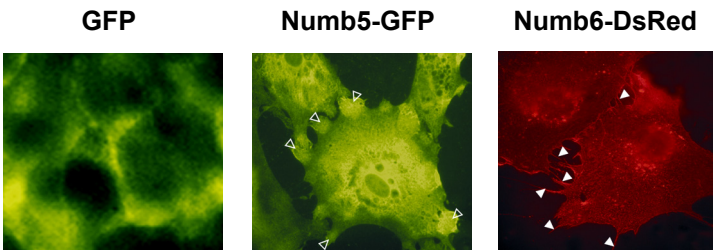

B

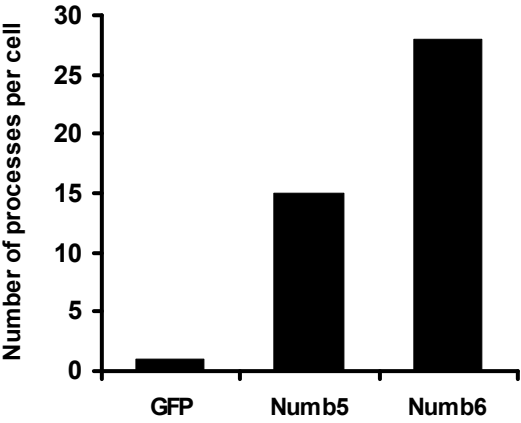

C

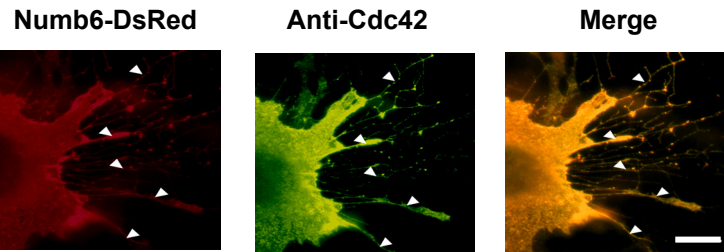

D

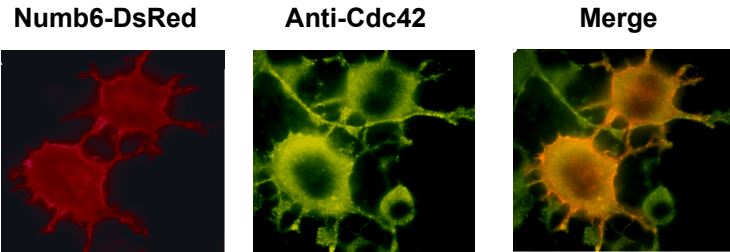

Supplement: Additional file 3 — Supplemental Figure 3. NUMB5 and NUMB6 enhance lamellipodial and filopodial growth. (A) NUMB5 and NUMB6 over-expression in neural progenitors enhances lamellipodial and filopodial extension, respectively. Arrowheads indicate lamellipodia; white arrowheads indicate filopodia. (B) Corresponding quantification of protrusion number in GFP, NUMB5, and NUMB6 overexpressing cells. (C) Confocal imaging shows that NUMB6 co-localizes with CDC42 in neural progenitors. NUMB6-DsRed infection, green CDC42 antibody staining, yellow, both merged. White arrowheads indicate filopodia. Scale bar = 5 μm. (D) Using confocal imaging, NUMB6 is co-localized with CDC42 in N2a cells. NUMB6-DsRed infection, green CDC42 antibody staining, yellow, both merged. [file 1749-8104-5-31-S3.PDF]
