# Supplementary material for: Two novel human NUMB isoforms provide a potential link between development and cancer
Source: Neural Dev. 2010 Dec 1;5:31. doi: 10.1186/1749-8104-5-31 (PMC3009962; doi:10.1186/1749-8104-5-31)
Supplement: Additional file 2 — Supplemental Table 1. Peptides found in tryptic digests of Numb isoform 5 and 6 electrophoretic bands as indicated in Figure 1D. [file 1749-8104-5-31-S2.PDF]

Table I Peptides found in tryptic digests of Numb isoform 5 and isoform 6 electrophoretic bands as indicated in Figure 1D.

peptide[a]	monoisotopic molecular mass (Da)		peptide[d]	monoisotopic molecular mass (Da)	
	protein band from NT2 cells	protein band from NTA cells		protein band from NT2 cells	protein band from NTA cells
QSFR	536.2776	536.2619	QSFR	536.2916	536.2950
GFFGK	554.2959	554.2853	VVDEK	588.3292	588.3191
VVDEK	588.3378	588.3119	TTFTR	624.3466	624.3039
TTFTR	624.3195	624.3231	LASADR		631.3559
EEIMK	648.3462		EEIMK		648.3249
CSFPVK	679.3430	679.3363	TFEIEL		750.3847
TFEIEL [b]	750.3626	750.3800	TPSEADR	774.3623	536.2705
AFSYICR	858.4478		AFSYICR	858.4340	858.3967
VSFCAPDR	893.4049	893.4065	WLEEVSK	889.4128	889.4545
GMHICEDAVK	1101.5090	1101.4947	VSFCAPDR	893.4089	893.4193
VTTATEQAER	1104.5089		VTTATEQAER		1104.5145
AVLWVSADGLR	1185.6023		DLIVDQTIEK		1172.6058
ECGVTATFDASR	1255.5115	1255.5503	ECGVTATFDASR		1255.5728
YLGHVEVDESR	1302.5899	1302.6204	TNPSPTNPFSSDLQK		1631.8584
LSHAVGCAFAACLER	1546.7059	1546.7385			
TNPSPTNPFSSDLQK [c]	1631.7648	1631.7791			

[a] Amino acid sequences matching the above peptides in the human Numb isoform 5, NCBI Protein Database accession no. ABY89090.1, in bold (underlined if adjacent sequences were mapped).

mnklr**qsfr**r kkdvyvpeas rphqwqtdee gvrtgk**csfp vkylghvevd esrgmhiced**
avkrlkaerk ffk**gffgk**tg kkavk**avlwv sadglrvvde k**tkdlivdqt iek**vsfcapd**
rnfdr**afsyi cr**dgttrrwi chcfmavkdt ger**lshavgc afaacler**kq krek**ecgvta**
tfdasrttft regsfr**vtta teqaereeim k**qmqdakkvp evegeaesis slcsqitnaf
stpedpfssa pmtkpvtvva pqsptfqgte wgqssgaasp glfqaghrrt pseadrwlee
vsksvraqqp qasaaplqpv lqppppptais qpaspfqgna fltsqpvpvg vvpalqpafv
paqsypvang mpypapnvpv vgitpsqmva nvfgtaghpq aahphqspsl vrqqtfphye
assattspff kppaqhlngs aafngvddgr lasadrhtev ptgtcpvdpf eaqwaalenk
skqr**tnpspt npfssdlqkt feiel**

[b, c] Sequences were confirmed by MS/MS.

[d] Amino acid sequences matching the above peptides in the human Numb isoform 6, NCBI Protein Database accession no. ABY89091.1, in bold (underlined if adjacent sequences were mapped).

mnklr**qsfr**r kkdvyvpeas rphqwqtdee gvrtgkcsfp vkylghvevd esrgmhiced
avkrlkatgk kavkavlwvs adglr**vvdek** tk**dlivdqti ekvsfcapdr** nfdr**afsyic**

rdgttrrwic hcfmavkdtg erlshavgca faaclerkqk rek**ecgvtat fdasrttftr**
egsfr**vttat eqaereeimk** qmqdakkvpe vegeaesiss lcsqitnafs tpedpfssap
mtkpvtvvap qsptfqgtew gqssgaaspg lfqaghrr**tp seadrwleev sk**svraqqpq
asaaplqpvl qppppptaisq paspfqgnaf ltsqpvpvgv vpalqpafvp aqsypvangm
pypapnvpvv gitpsqmvan vfgtaghpqa ahphqspslv rqqtfphyea ssattspffk
ppaqhlngsa afngvddgr**l asadr**htevp tgtcpvdpfe aqwaalenks kqr**tnpsptn**
pfssdlqktf eiel
